# The risk of pediatric bicycle handlebar injury compared with non-handlebar injury: a retrospective multicenter study in Osaka, Japan

**DOI:** 10.1186/s13049-015-0146-7

**Published:** 2015-09-17

**Authors:** Tomoya Hirose, Hiroshi Ogura, Takeyuki Kiguchi, Yasuaki Mizushima, Futoshi Kimbara, Junya Shimazaki, Shigeru Shiono, Hitoshi Yamamura, Akinori Wakai, Ryosuke Takegawa, Hisatake Matsumoto, Mitsuo Ohnishi, Takeshi Shimazu

**Affiliations:** Department of Traumatology and Acute Critical Medicine, Osaka University Graduate School of Medicine, 2-15 Yamadaoka, Suita, Osaka 565-0871 Japan; Department of Emergency Medicine, Osaka General Medical Center, 3-1-56 Bandai-Higashi, Sumiyoshi-ku, Osaka 558-8558 Japan; Senshu Trauma and Critical Care Center, Rinku General Medical Center, 2-23 Rinku-orai kita, Izumisano, 598-8577 Japan; Senri Critical Care Medical Center, Osaka Saiseikai Senri Hospital, 1-1-6 Tsukumodai, Suita, Osaka 565-0862 Japan; Emergency and Critical Care Medical Center, Osaka Police Hospital, 10-31 Kitayama-cho, Tennouji-ku, Osaka 543-0035 Japan; Osaka Prefectural Nakakawachi Medical Center of Acute Medicine, 3-4-13 Nishiiwata, Higashiosaka City, Osaka 578-0947 Japan; Department of Trauma and Critical Care Medicine, Osaka City University Graduate School of Medicine, 1-4-3 Asahimachi, Abenoku, Osaka 545-8585 Japan; Traumatology and Critical Care Medical Center, National Hospital Organization Osaka National Hospital, 2-1-14 Hoenzaka Chuo-ku, Osaka, Osaka 540-0006 Japan

## Abstract

**Background:**

Bicycle accidents are one of the major causes of unintentional traumatic injury in childhood. The purpose of this study was to examine characteristics and risks of handlebar injury in childhood.

**Methods:**

We conducted a more than 5-year retrospective survey of patients under 15 years of age with bicycle-related injuries admitted to eight urban tertiary emergency centers in Osaka, Japan. Patients were divided into the direct-impact handlebar injury (HI) group and the non-handlebar injury (NHI) group.

**Results:**

The HI group included 18 patients and the NHI group included 308 patients. Median Injury Severity Score (ISS) in the HI group was 9. Injury sites included the chest, 2 (chest bruise, 1; tracheal injury, 1) and abdomen, 16 (hepatic injury, 6; pancreatic injury, 2; duodenal injury, 1; splenic injury, 1; small intestinal injury, 1; retroperitoneal hemorrhage, 1; renal injury, 1; abdominal wall musculature injury, 2; bladder injury, 1; and perineal laceration, 1). There were no significant differences in age, sex, ISS, and prognosis between the two groups. However, significant differences were seen in the abdominal median Abbreviated Injury Scale (AIS) score, which was higher in the HI group (3 vs 0, *p* < 0.01), and in the head median AIS score, which was higher in the NHI group (0 vs 2, *p* < 0.01). As mechanisms of injury, falling while riding a bicycle occurred significantly more frequently in the HI group (17 [94.4 %] vs 65 [21.1 %], *p* < 0.01). Direct transportation from the scene of the accident occurred significantly more often in the NHI group (5 [27.8 %] vs 255 [82.8 %], *p* < 0.01), whereas transfer from another hospital occurred significantly more frequently in the HI group (11 [61.1 %] vs 45 [14.6 %], *p* < 0.01).

**Conclusions:**

Handlebar injuries in children have significant potential to cause severe damage to visceral organs, especially those in the abdomen.

## Background

Bicycle accidents are one of the major causes of unintentional traumatic injury in childhood; the number of incidents in children under 15 years of age was 26,245 in 2011 in Japan [1]. Children riding bicycles have a higher risk of accidents compared with adults because children have fewer rules and engage in more risky behavior than adults [1]. Helmet use in children has been promoted for the prevention of head injury from bicycle accidents [2, 3]. However, the risk of injuries from direct impact with handlebars has not generally been recognized among children [4, 5]. Therefore, the purpose of this study was to examine the characteristics and risks of childhood handlebar injury compared with non-handlebar injury in Japan.

## Methods

### Patients and setting

**Fig. 1:**
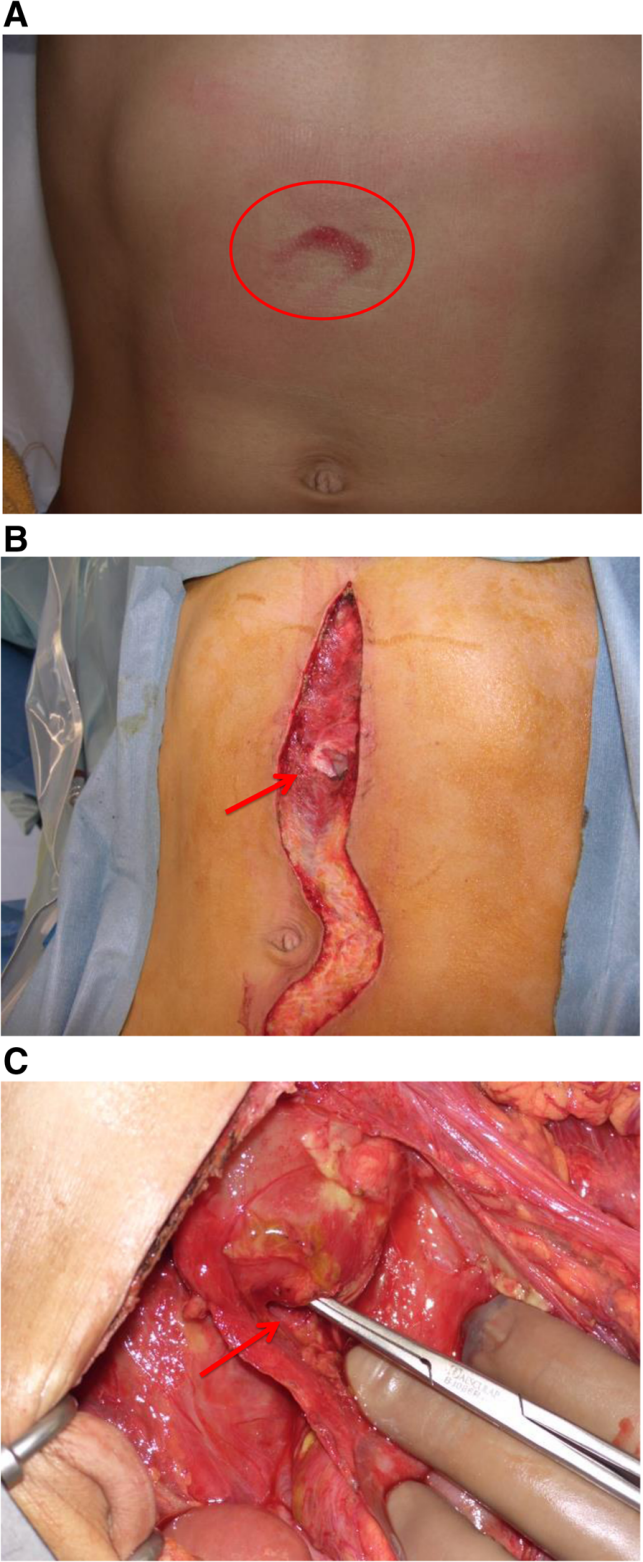
Photographs of a patient who sustained a handlebar injury. **a** A typical skin bruise (*within circle*) from a handlebar injury was suggestive of damage to the abdominal organs. **b**, **c** We diagnosed the patient as having injury to the abdominal wall musculature (**b**, *arrow*) and duodenum (**c**, *arrow*). Surgical treatment was required to repair these injuries

This study was a more than 5-year retrospective survey of patients under 15 years of age with bicycle-related injuries admitted to eight urban tertiary emergency centers in the Osaka area of Japan and was approved by the Ethics Committee of the Osaka University Graduate School of Medicine. We chose the pediatric bicycle injury patients admitted to each institution and collected patient information from their medical records. We identified cases of bicycle-related injury retrospectively by checking the medical records of all trauma patients under 15 years of age. The period for which each hospital had patients enrolled ranged from 5 years (2008–2012) to 12 years (2000–2012). Patients were divided into the direct-impact handlebar injury (HI) group and the non-handlebar injury (NHI) group. Patients with a medical record description of “handlebar injury” or “skin bruise to the body from a handlebar injury” were assigned to the HI group. A typical skin bruise from the handlebar injury is shown in Fig. 1. Patients with other non-handlebar-impact bicycle-related injuries, such as falling while riding a bicycle or collision with vehicles, were assigned to the NHI group. We assessed age, sex, Injury Severity Score (ISS), prognosis, mechanisms of injury, Abbreviated Injury Scale (AIS) score, AIS score of 3 or greater, Glasgow Coma Scale (GCS) on hospital arrival, treatment, medical transport method, and time from accident to arrival at our emergency centers in each patient and compared these variables between the two groups. The AIS score was determined retrospectively for this study, and the GCS on hospital arrival was prospectively given in the records.

### Statistical analysis

Continuous variables are presented as the median and interquartile range (IQR). The Wilcoxon rank-sum test and Fisher’s exact test were used to compare two patient groups. A *p* value of < 0.05 was considered significant. All statistical analyses were performed using JMP 9.0.2 (SAS Institute Inc., Cary, NC, USA).

## Results

Patient characteristics are shown in Table 1. The HI group included 18 patients, and the NHI group included 308 patients. In the HI group, the prognosis was good in all patients. Injury sites were as follows: chest, 2 (chest bruise, 1; tracheal injury, 1); abdomen, 16 (hepatic injury, 6; pancreatic injury, 2; duodenal injury, 1; splenic injury, 1; small intestinal injury, 1; retroperitoneal hemorrhage, 1; renal injury, 1; abdominal wall musculature injury, 2; bladder injury, 1; and perineal laceration, 1) (Table 2, Fig. 1). Therapeutic interventions were performed in 9 patients and included emergency surgery or emergency transcatheter arterial embolization (TAE) in 6 and elective surgery or elective TAE in 3 patients. The other 9 patients were followed with close observation (Tables 1 and 2). There were no significant differences in age, sex, ISS, and prognosis between the two groups (Table 1).

**Table 1:** Patient characteristics

	Handlebar impact group	Non-handlebar impact group	*p* value
Number	18	308	
Age (IQR)	9 (7–13)	11 (7–14)	0.288
Male (%)	14 (77.8)	209 (67.9)	0.446
Injury Severity Score (IQR)	9 (4–10)	9 (5–17)	0.252
ICU stay (days) (IQR)	5.5 (2–7)	2 (0–4)	0.004
Hospital stay (days) (IQR)	10.5 (6.75–30.5)	5 (2–14)	0.016
GCS on arrival (IQR)	15 (15–15)	15 (13–15)	0.005
Shock on arrival (%)	1 (5.6)	10 (3.2)	0.470
Survivors (%)	18 (100)	297 (96.4)	1.000
Treatment (%)			
Emergency surgery/TAE	6 (33.3)	75 (24.4)	0.4043
Elective surgery/TAE	3 (16.7)	20 (6.5)	0.1245
Conservative treatment	9 (50 %)	215 (69.8)	0.1134

*IQR* Interquartile range, *ICU* Intensive care unit, *GCS* Glasgow Coma scale, *TAE* Transcatheter arterial embolization

**Table 2:** Characteristics of patients in the handlebar impact group

Age	Sex	Injuries	ISS	Time from injury to final hospital	Treatment
9	M	Renal injury	16	5 h	Emergency TAE
12	M	Hepatic injury	10	26 min	Emergency TAE
15	M	Splenic injury	17	15 min	Emergency TAE
5	M	Pancreatic injury	9	48 h	Emergency surgery
14	M	Duodenal injury, abdominal wall musculature	10	210 min	Emergency surgery
9	F	Small intestinal injury	10	3 h	Emergency surgery
13	M	Bladder rupture, abdominal wall musculature	17	3 h	Elective surgery
14	M	Pancreatic injury	5	12 h	Elective surgery
7	M	Hepatic injury	9	6 h	Elective TAE
6	F	Perineal laceration	2	3 h	Conservative treatment (suture only)
8	M	Hepatic injury	2	3 h	Conservative treatment
9	M	Hepatic injury	4	3 h	Conservative treatment
3	M	Hepatic injury	9	75 min	Conservative treatment
13	M	Hepatic injury	4	3 h	Conservative treatment
7	M	Tracheal injury, mediastinal emphysema	10	3 h	Conservative treatment
11	M	Retroperitoneal hematoma	9	15 min	Conservative treatment
10	M	Abdominal bruise	1	191 min	Conservative treatment
7	M	Chest bruise	1	Unknown	Conservative treatment

*ISS* Injury severity score, *TAE* Transcatheter arterial embolization

However, significant differences were seen in the abdominal median AIS, which was higher in the HI group compared with NHI group, and in the number of patients with abdominal AIS score of 3 or greater, which was also significantly higher in the HI group. The head median AIS score was significantly higher in the NHI group, and the number of patients with a head AIS score of 3 or greater was also significantly higher in the NHI group (Table 3). The GCS on hospital arrival was significantly lower in the patients in the NHI group (GCS score 15: *n* = 176, 14: *n* = 50, 13: *n* = 17, 12: *n* = 8, 11: *n* = 9, 10: *n* = 4, 9: *n* = 8, 8: *n* = 5, 7: *n* = 7, 6: *n* = 5, 5: *n* = 5, 4: *n* = 4, 3: *n* = 10) than in those in the HI group (GCS score 15: *n* = 16, 14: *n* = 2) (Table 1).

**Table 3:** Abbreviated Injury Scale (AIS) score and AIS score of ≥3 in the handlebar impact versus non-handlebar impact group

	Handlebar impact group	Non-handlebar impact group	*p* value
Number of patients	18	308	
AIS score (median)			
Head (IQR)	0 (0–0)	2 (0–4)	<0.001
Face (IQR)	0 (0–0)	0 (0–0)	0.099
Chest (IQR)	0 (0–0)	0 (0–0)	0.278
Abdomen (IQR)	3 (1.75–3)	0 (0–0)	<0.001
Pelvic & extremity (IQR)	0 (0–0)	0 (0–0)	0.025
Soft tissue (IQR)	1 (0–1)	1 (0–1)	0.171
AIS ≥3 (n)			
Head (%)	0 (0)	130 (42.2)	<0.001
Face (%)	0 (0)	1 (0.3)	1.000
Chest (%)	1 (5.6)	42 (13.6)	0.486
Abdomen (%)	11 (61.1)	19 (6.2)	<0.001
Pelvic & extremity (%)	0 (0)	27 (8.8)	0.381
Soft tissue (%)	0 (0)	0 (0)	1.000

*IQR* Interquartile range

Regarding mechanisms of injury, falling while riding a bicycle occurred significantly more frequently in the HI group, whereas the incidence of collision with vehicles was significantly higher in the NHI group (Table 4). Direct transportation from the scene of the accident to our emergency centers occurred significantly more often in the NHI group, whereas transfer to our emergency centers from another hospital occurred significantly more frequently in the HI group (Table 4). The time from accident to arrival at our emergency centers was significantly longer in the HI group than in the NHI group (Table 4).

**Table 4:** Mechanism of injury, medical transport method, and time from accident to hospital in the handlebar impact versus non-handlebar impact group

	Handlebar impact group	Non-handlebar impact group	*p* value
Number of patients	18	308	
Mechanism of injury			
Single bicycle accident (%)	18 (100)	76 (24.7)	<0.001
Fall from bicycle (%)	17 (94.4)	65 (21.1)	<0.001
Collision with obstacle (%)	1 (5.6)	11 (3.6)	0.500
Contact accident with car or motorcycle (%)	0 (0)	232 (75.3)	<0.001
Transport			
Ambulance/helicopter (%)	5 (27.8)	255 (82.8)	<0.001
Walk-in (%)	2 (11.1)	8 (2.6)	0.010
Hospital transfer (%)	11 (61.1)	45 (14.6)	<0.001
Time to hospital (min)	180 (127.5–255) *n* = 17	34 (26–50) *n* = 276	<0.001

## Discussion

The numbers of bicycles and associated riders have increased, and bicycle-related injuries have become a major health problem [6]. Bicycle trauma comprises a significant proportion of trauma in children. However, the risk of pediatric bicycle handlebar injury has not been emphasized. In 1997 in the United States, 1.15 per 100,000 subjects 19 years and younger were estimated to have suffered serious abdominal and pelvic organ injury leading to hospitalization that was associated with non-motor-vehicle bicycle handlebar accidents [5]. Winston et al. [7] considered handlebars as hidden spears because impact with handlebars might be accompanied by visceral organ injury through the concentration of an external force applied by the end of the handlebar to a child’s body.

The typical mechanism of pediatric bicycle handlebar injury is a falling accident in which the child loses control of the bicycle, begins to fall, the front wheel turns to the side, and the end of the bicycle handlebar strikes the neck, chest, abdomen, or pelvic area of the rider [7, 8]. We consider that pediatric bicycle handlebar injury can be caused by the immature decision-making ability of the child, the impact caused by sudden braking or collision, and the insufficient muscular power of the child’s body to withstand such impacts. About 90 % of patients with significant intra-abdominal organ injury were reported to have visible skin bruises from handlebar contact [9, 10] such as that shown in Fig. 1a.

Direct-impact handlebar injuries to the liver, spleen, pancreas, duodenum, intestines, kidney, urethra, abdominal wall, and major vessels have been reported [4, 8]. The rate of handlebar injuries to parenchymatous organs such as the liver, kidney, pancreas, and spleen reportedly ranges from 20 to 37 % [4, 11–13], and gastrointestinal perforation has been reported in 9–10 % of children with handlebar injuries [4, 11, 12]. Cevik et al. [10] reported that 85.7 % of children who sustained direct-impact handlebar injuries required operative intervention. In our study, 50.0 % of the children who sustained direct-impact handlebar injuries required operation or TAE intervention (Tables 1 and 2). These are surprisingly high rates, which indicate that children with handlebar injuries should be examined carefully.

Traumatic abdominal wall hernia is a well-known complication of handlebar injuries and is defined as herniation through disrupted musculature and fascia associated with blunt trauma, without skin penetration because of the skin’s elasticity, and with no evidence of prior hernia defect at the site of injury [4, 14, 15]. However, traumatic abdominal wall hernia is rare, so due to a lack of knowledge of this condition, apparent clinical signs associated with this injury might easily be missed [16]. Two of the patients in the present study had injuries to their abdominal wall musculature and required surgical repair (Table 2, Fig. 1). Knowledge of the mechanism of trauma to the abdomen and visible handlebar skin bruises, if they exist, can help the physician to suspect the presence of traumatic abdominal wall trauma.

Falling while riding a bicycle occurred significantly more frequently and GCS on hospital arrival was significantly higher in the HI group compared with the NHI group (Tables 1 and 4). It is possible that the severity of pediatric handlebar injuries is underestimated by parents, witnesses, and the ambulance crew at the scene of an accident. Thus, transfer from another hospital occurred significantly more frequently and the time from accident to patient arrival at our emergency centers was significantly longer in the HI group (Table 4). In our study, one patient with pancreatic injury was not transferred to our center until 48 h after the injury (Table 2). The severity of pediatric bicycle handlebar injury might often be underestimated when based on the mechanism of the accident or the level of consciousness of the patient. We suggest more liberal use of CT scanning in the assessment of severe handlebar injury, at least for those injuries involving the abdomen.

There are some limitations in this study. First, it is a retrospective study. We collected patient information only from patient medical records. Second, study periods are different for each medical institution because medical records must be preserved for at least 5 years in Japan. Third, the medical institutions participating in this research were all urban tertiary emergency centers in Osaka, Japan. In major metropolitan areas of Japan, hospitals are categorized into three levels of emergency care (Primary emergency care: for patients with low-acuity conditions who can be safely discharged home; Secondary emergency care: for patients with moderate-acuity conditions who require admission to a regular inpatient bed; and Tertiary emergency care: for patients with high-acuity conditions who require admission to the ICU). Therefore, the subjects of this study were urban residents, and they had been judged as having severe or suspected severe trauma at the accident scene or initial medical institution before they were transported to our centers. This might lead to the difference in sample size of the HI and NHI groups in the present study. The research data does not cover all pediatric bicycle accidents. More comprehensive research into pediatric bicycle injuries would be desirable in a future study. Fourth, adult patients with handle bar injury were not included in this study. There is little documentation on bicycle handle bar injury in adult patients because of the following possible reasons: i) the abdominal muscles of adults are more well developed than those of children, ii) adults are less inclined to ride bicycles as recklessly as children do, and iii) adults generally can better perceive risks than children can [17].

As a preventive strategy for traumatic handlebar injury in the future, it might be effective to modify the shape of the ends of the handlebars, limit the side-to-side rotation of the front wheel fork assembly, and promote the wearing of an abdominal protector to prevent pediatric handlebar injuries.

## Conclusions

It should be emphasized that handlebar injuries in children have a significant potential to cause severe damage to visceral organs, especially those in the abdomen. Such injuries require a high degree of suspicion so that visceral organ damage from handlebar injuries can be detected early and appropriate treatment can be administered.
